# Photo Crosslinkable Hybrid Hydrogels for High Fidelity Direct Write 3D Printing: Rheology, Curing Kinetics, and Bio-Scaffold Fabrication

**DOI:** 10.3390/jfb17010030

**Published:** 2026-01-04

**Authors:** Riley Rohauer, Kory Schimmelpfennig, Perrin Woods, Rokeya Sarah, Ahasan Habib, Christopher L. Lewis

**Affiliations:** 1 Department of Biomedical Engineering, Rochester Institute of Technology, Rochester, NY 14623, USA; rbr9577@rit.edu; 2 Department of Chemical Engineering, Rochester Institute of Technology, Rochester, NY 14623, USA; kms6060@rit.edu; 3 Department of Manufacturing and Mechanical Engineering Technology, Rochester Institute of Technology, Rochester, NY 14623, USA; pcw3635@rit.edu; 4 Sustainable Product Design and Innovation, Keene State College, Keene, NH 03435, USA; rokeya.sarah@keene.edu

**Keywords:** direct-write 3D bioprinting, hybrid hydrogels, tunable properties, UV crosslinking, filament fusion

## Abstract

This work characterizes hybrid hydrogels prepared via the combination of natural and synthetic polymers. By incorporating a biocompatible compound, poly(ethylene glycol) diacrylate (PEGDA, M_n_ = 400), into alginate and carboxymethyl cellulose (CMC)-based hydrogels, the in situ UV crosslinking of these materials was assessed. A custom direct-write (DW) 3D bioprinter was utilized to prepare hybrid hydrogel constructs and scaffolds. A control sample, which consisted of 4% *w*/*v* alginate and 4% *w*/*v* CMC, was prepared and evaluated in addition to three PEGDA (4.5, 6.5, and 10% *w*/*v*)-containing hybrid hydrogels. Rotational rheology was utilized to evaluate the thixotropic behavior of these materials. Filament fusion tests were employed to generate bilayer constructs of various pore sizes, providing metrics for the printability and diffusion rate of hydrogels post-extrusion. Printability indicates the shape fidelity of pore geometry, whereas diffusion rate represents material spreading after deposition. Curing kinetics of PEGDA-containing hydrogels were evaluated using photo-Differential Scanning Calorimetry (DSC) and photorheology. The Kamal model was fitted to photo-DSC results, enabling an assessment and comparison of the curing kinetics for PEGDA-containing hydrogels. Photorheological results highlight the increase in hydrogel stiffness concomitant with PEGDA content. The range of obtained complex moduli (G*) provides utility for the development of brain, kidney, and heart tissue (620–4600 Pa). The in situ UV irradiation of PEGDA-containing hydrogels improved the shape fidelity of printed bilayers and decreased filament diffusion rates. In situ UV irradiation enabled 10-layer scaffolds with 1 × 1 mm pore sizes to be printed. Ultimately, this study highlights the utility of PEGDA-containing hybrid hydrogels for high-resolution DW 3D bioprinting and potential application toward customizable tissue analogs.

## 1. Introduction

Tissue engineering addresses the shortage of tissue and organ scaffolds for regenerative medicine, finding applications in organ transplantation research, bone tissue, and tracheal tissue repair [1,2]. Extrusion-based 3D bioprinting is unique in its ability to precisely deposit natural and synthetic polymers, or bioinks, with high cell viability compared to other methods of bioprinting [3,4]. Controlled deposition enables the construction of complex scaffolds capable of mimicking patient tissue structures, expanding the field of tissue engineering and personalized medicine [5,6,7]. The selection of a material for extrusion-based bioprinting is based upon its capacity to exhibit shear thinning, where viscosity is reduced under the application of shear force [8]. Not all shear-thinning materials are suitable, as the bioinks must have sufficient post-print stability, i.e., the filament must be able to hold shape immediately post-extrusion [8,9]. This characteristic green strength is necessary for the longevity and structural integrity of the print during the incubation period [10]. As such, the required rheological properties, such as recovery rate, controlled shear thinning behavior, and optimal storage modulus, are critical factors to successfully prepare a tissue construct. Bioinks that strike a balance between these properties enable the development of 3D printed high-resolution scaffolds with biological functionality.

To accomplish this, composite inks are developed by combining synthetic and natural materials to obtain hybrid hydrogels with desirable characteristics for 3D bioprinting [11,12,13]. Hydrogels represent an important class of materials for biomedical applications, where water uptake promotes polymer chain motion and special functionality, similar to biotissue [14]. Materials with known properties, such as alginate [15,16], carboxymethyl cellulose (CMC) [12,17], chitosan [18,19], collagen [20,21], various nanofibers [22,23], and gelatin [24,25], are frequently combined to create hydrogels with controlled shear thinning behavior, high recovery rates post-extrusion, and storage moduli that match parent biological tissue structures.

In our previous work [17,26], a novel bioink comprising alginate and CMC was introduced, providing evidence that a combination of these materials can enhance geometric fidelity (≤20% deviation in pore geometry) as well as cell viability and proliferation with pancreatic cancer cells (BxPC3); this work established that the optimal formulation of 4% alginate and 4% CMC through extensive characterization via cytotoxicity tests. In particular, a hybrid alginate–CMC bioink (A_4_C_4_) encapsulating BxPC3 pancreatic cancer cells achieved ~86% viability after 23 days of culture [17]. Moreover, when bioprinted at a low extrusion pressure (~8 psi), A_4_C_4_ scaffolds maintained ≈90% cell viability after 15 days [26]. This high long-term viability was consistent across multiple cell lines: for example, prostate cancer cells and HEK-293 kidney cells showed ~89% and ~87% viability, respectively, after 15 days in A_4_C_4_ constructs [26]. These results indicate that the A_4_C_4_ base material provides a cytocompatible 3D matrix conducive to cell survival and proliferation over weeks. While initial research relied on an ionic crosslinker (CaCl_2_), in order to combat the poor mechanical properties of alginate and CMC [27], the current work investigates the effects of photo-crosslinking by adding various concentrations of poly(ethylene glycol) diacrylate (PEGDA), and a constant concentration of photoinitiator lithiumphenyl-2,4,6-trimethylbenzoylphosphinate (LAP) to augment the optimized hydrogel formulation. The photo-crosslinkable compound, PEGDA, was selected based on its biocompatibility, mechanical properties, and previous studies on its positive interactions with alginate [28,29,30,31,32]. The incorporation of PEGDA to prepare novel hybrid hydrogel formulations can improve printability (P_R_) and cellular activity by enabling the generation of more precise scaffolds, owing to its ability to cure rapidly upon exposure to UV light.

Shape retention following direct-write (DW) 3D bioprinting is a challenge, where hydrogels tend to flow post-print, which results in poor shape fidelity; we hypothesize that the incorporation of photo-crosslinkable monomer PEGDA will enable high shape fidelity and the preparation of more complex geometries. In this investigation, a set of bioinks comprising various concentrations of PEGDA (4.5, 6.5, and 10% *w*/*v*), LAP, alginate, and CMC was thoroughly characterized. Rotational rheometry was employed to understand the materials recovery rate and shear thinning parameters prior to print tests. These formulations were subjected to photorheological and photo-DSC tests to enable an assessment of the curing kinetics and behavior, providing insights that could be translated to establish printing parameters for use in subsequent printing tests. A custom-built 3D bioprinter equipped with in situ dual-crosslinking capabilities was used to characterize the printability of each bioink via a series of tests designed to examine shape fidelity, resolution, and post-extrusion mechanical properties; these tests included filament fusion [9], filament collapse [9], swelling assessments [33], and the fabrication of multi-layered scaffolds with defined pore structures. This study underscores the importance of UV rheological characterization in predicting real-time material behavior during bioprinting. Understanding curing kinetics in conjunction with the material’s viscosity recovery rate allows researchers to evaluate each compound’s crosslinking behavior prior to performing any print test. Additionally, the augmentation of hydrogels with PEGDA provides a facile approach to tuning material properties. This methodology results in a more efficient development process for bioinks, as researchers can fine-tune their print settings and select the correct application for each material based on its curing kinetics and photorheological factors.

## 2. Materials and Methods

### 2.1. Materials

Alginic acid sodium salt from brown algae (alginate, medium viscosity) and carboxymethylcellulose sodium salt (CMC, medium viscosity) were purchased from Sigma-Aldrich (St. Louis, MO, USA). Poly(ethylene glycol) diacrylate (PEGDA, Mn = 400 g/mol) was obtained from Polysciences (Warrington, PA, USA). Lithiumphenyl-2,4,6-trimethylbenzoylphosphinate (LAP, Mw = 294.21 g/mol) was obtained from Sigma-Aldrich (St. Louis, MO, USA). The chemical structures of these materials are shown in Supplemental Figure S1.

### 2.2. Hydrogel Preparation

To prevent premature crosslinking, all hydrogels were prepared and stored in a UV light-restricted environment. Three batches of 15–30 mL containing 4% *w*/*v* alginate, 4% *w*/*v* CMC, 0.1% *w*/*v* LAP, and varying concentrations of PEGDA (4.5, 6.5, and 10% *w*/*v*) were mixed for all testing and printing. Additionally, a control sample containing 4% *w*/*v* alginate and 4% *w*/*v* CMC was prepared. A concentration of 0.1% (*w*/*v*) LAP was determined based on the toxicity ranges of an investigation by Xu et al. [29]. These formulations are referred to as H-XX, where XX represents the percent *w*/*v* of PEGDA.

As an example, H-6.5 was prepared as follows: 0.015 g LAP was dissolved in 12.8 g deionized H_2_O by overhead mechanical stirring at 150 rpm for 5 min. Alginate (0.6 g, 4% *w*/*v*) and CMC (0.6 g, 4% *w*/*v*) were weighed out and placed in a separate beaker, followed by the addition of 0.98 g PEGDA via borosilicate pipette to the LAP/H_2_O solution. Subsequently, the LAP/H_2_O/PEGDA solution was allowed to stir at 150 rpm for an additional 5 min. Lastly, the alginate/CMC mixture was slowly added to the LAP/H_2_O/PEGDA solution and stirred via overhead mechanical stirrer in two 15 min intervals to obtain homogeneous hydrogels.

### 2.3. Rheological Analysis

Rotational rheological measurements (Discovery HR2 Hybrid Rheometer, TA Instruments, New Castle, DE, USA) were obtained at room temperature (20 °C) using a cone and plate geometry (20 mm). A steady rate sweep was performed at shear rates ranging from 1 to 1000 s^−1^ to determine viscosity and shear thinning parameters. A MATLAB (R2018b) script was utilized to fit the data to the power-law model (Equation (1)) to obtain shear thinning coefficients *n* and *K* with high accuracy (*R*^2^ > 99%), ensuring the reliability of this model in describing the hydrogels’ rheological properties.

(1) $$\eta =K{\dot{\gamma }}^{n-1}$$

Here, viscosity and shear rate are represented by η and $\dot{\gamma }$, respectively. Additionally, a three-interval thixotropy test (3ITT) was performed to assess each material’s ability to recover its viscosity post-printing. Samples were subjected to a shear rate of 0.1 s^−1^ for 120 s to establish a baseline, after which the shear rate was increased to 600 s^−1^ for 60 s to mimic extrusion conditions and subsequently returned to 0.1 s^−1^ for 120 s. The recovery rate (%) provides insight as to how quickly the material can recover its baseline viscosity after being subjected to a high shear rate and was calculated via Equation (2):

(2) $$recovery\;rate=\;\frac{\eta_{t}}{\eta_{120s}}\times 100$$

Here, $\eta_{t}$ is viscosity during the third interval and $\eta_{120s}$ is the viscosity obtained for the first interval (taken at 120 s).

### 2.4. 3D Bioprinting and Scaffold Fabrication

Printing tests were conducted on a custom in-house 3-axis “bedslinger” bioprinter, equipped with an in situ dual (physical, chemical) crosslinking system. This system is shown in Supplemental Figure S2. The bioinks were loaded into an amber Syringe Barrel (Nordson, EFD) and pneumatically dispensed through an opaque tapered syringe tip (Sanants, 0.017″/0.43 mm tip) onto a glass print bed. UltiMaker CURA 5.7.2 with a custom post-processing plugin was utilized to slice all printed geometries not written by hand. Print tests, including variations in single lines and bilayer scaffolds, were performed to characterize the behavior of the material. To maintain good biocompatibility and cell health, the UV range of 365–405 nm is employed [29]; for this investigation, all irradiation was completed at 365 nm. In addition to meeting the wavelength criteria, the photoinitator had to be water-soluble, biocompatible, and have proven use within bioprinting. LAP was chosen over the more commonly used Irgacure 2959 based on the results of a study performed by Heqi Xu et al. [29], indicating that LAP exhibited superior water solubility, toxicity, biocompatibility, and curing kinetics at the specified wavelengths.

### 2.5. Filament Shape Fidelity Test

A filament shape fidelity test was run per composition to evaluate their extrusion behavior at varying extrusion pressures of 12, 14, 16, and 18 psi. Tests were run at 5 mm/s and were photographed immediately after printing to be measured with ImageJ 1.54p It is expected that some degree of die swell occurs immediately following extrusion, upon entering free flow. Following deposition, material relaxation occurs. The quantification of this phenomenon is captured via filament shape fidelity (%), which is obtained by calculating the ratio between the measured filament diameter ($\emptyset_{m}$) and nozzle diameter (0.43 mm) values.

(3) $$filament\;shape\;fidelity\;\left(\%\right)=\left(1-\frac{\emptyset_{m}-0.43}{0.43}\right)\times 100$$

Additionally, this test can be used to observe continuity of the filament, providing insight into printability and consistency at varying extrusion pressures.

### 2.6. Printability and Shape Fidelity Test

The test was performed as described by Habib et al. [17], and a short summary of it is written here. Three uncrosslinked bilayer scaffolds with varying pore sizes were printed at 5 mm/s to determine the diffusion rate (rate of material spreading) (Df_R_) and printability (P_R_) of the compounds, using equations Equations (4) and (5), respectively. The scaffolds had an increasing filament distance of 1–5 mm with a 1 mm increment. A photo of the fabricated scaffold was taken from above immediately post-printing for analysis and measured using ImageJ.

(4) $$Df_{R}=\frac{A_{t}^{f}-A_{a}^{f}}{A_{t}^{f}}$$

(5) $$P_{R}=\frac{L^{2}}{16A_{a}^{f}}$$

where $A_{t}^{f}$ and $A_{a}^{f}$ are the theoretical and actual areas of the pore, respectively, and *L* is the perimeter of the actual pore. When there is no material spreading the diffusion rate is zero, as $A_{t}^{f}$ = $A_{a}^{f}$, and a perfectly square pore would have a printability of one. All measurements and calculated values are representative of an average sample size of three per composite.

### 2.7. Photo-Differential Scanning Calorimetry (DSC)

A TA Instruments DSC-250 equipped with a photocuring attachment (PCA) and an Omnicure S2000 UV light source (Excelitas Technologies, Pittsburgh, PA, USA) (365 nm) was used. Prior to each experiment, the light source was calibrated to ensure sample light intensity exposure remained a constant 10–11 mW/cm^2^. Each experiment was performed according to the following program using a 5–6 mg sample: (1) equilibrate and hold the cell temperature at 20 °C for 30 s, (2) expose the cell to UV light for 70 s, and (3) hold the cell temperature at 20 °C for 60 s.

### 2.8. Photorheology

A TA Instruments Discovery HR-20 rheometer equipped with a UV curing attachment was used in this investigation. An Omnicure S2000 containing a filter (λ = 365 nm) was used as the light source. Calibration of the source was performed using a UV Design Silver Line All UV-UV radiometer (Brachttal, Germany), with calibrations performed at 6 mW/cm^2^ and 20 °C. Oscillatory strain measurements were carried out at a frequency of 0.1 Hz, a fast-sampling strategy (2 points s^−1^), a gap size of 100 µm, and with the use of auto-strain adjustment to ensure a high signal-to-noise ratio across the spectrum of material properties. Linear viscoelasticity was confirmed for a representative material in both the uncured and cured states through a strain amplitude test. To account for volumetric changes that occur during curing, the normal force during testing was maintained at 0 N (0.1 N sensitivity). Each experiment was performed according to the following program: the lamp shutter was closed for the first 30 s of the test, opened for 15 s, and then closed again, where data was collected for an additional 30 s following irradiation to observe convergence to an equilibrium condition.

### 2.9. Freeform Scaffold

To test the layering abilities and construct fidelity of the composites containing 6.5% and 10% PEG-DA, a ten-layer construct, crosslinked in situ with 365 nm of UV light, was fabricated for both. All scaffolds were printed at 14 psi, 5 mm/s, and 5 mW/cm^2^ with a pore size of 1 mm. The first five layers of the construct were a 16 × 16 grid, followed by five layers of an 8 × 8 grid overtop. A summary of these parameters is shown in Table 1.

### 2.10. Cytotoxicity Test

Human mesenchymal stem cells (MSCs) were seeded onto standard tissue culture plastic (TCP). Three experimental conditions were established to evaluate cell–material interactions: (1) MSCs cultured directly on TCP, (2) MSCs on TCP with 0.5 g of hydrogel gently overlaid onto the adhered cells, and (3) MSCs seeded directly onto a pre-cast hydrogel layer formed on the TCP surface. All samples were maintained in standard MSC growth media and incubated for 24 h. Following incubation, brightfield microscopy was used to assess general cell morphology, while live/dead fluorescence staining was performed to evaluate short-term cell viability.

## 3. Results

### 3.1. Shear-Thinning and Thixotropic Behavior

**Figure 1:**
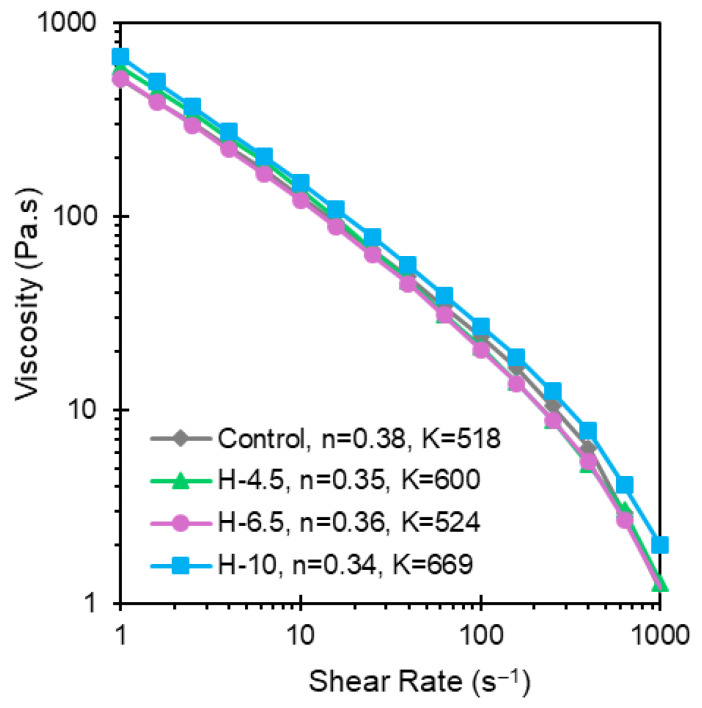
Flow analysis of hybrid hydrogels composed of 4% alginate/4% CMC, and 4.5, 6.5, and 10% PEGDA. The viscosity plot demonstrates shear-thinning characteristics, with a significant drop in viscosity as shear rate increases for all formulations.

The shear-thinning behavior of hydrogels composed of 4% alginate, 4% CMC, 0.1% LAP, and various concentrations of PEGDA (4.5%, 6.5%, and 10%) was evaluated via rotational rheometry as shown in Figure 1. Comparing the calculated power law parameters *K* and *n* provides insight into the printability of each formulation and therefore motivates which formulations are well-suited for extrusion-based 3D bioprinting.

*K* and *n* represent consistency and flow behavior index, respectively. As shown in Figure 1, the control exhibits the lowest consistency index (*K*) of all the formulations tested, with values of *K* and *n* that agree well with our previous investigation [26]. There is a decrease in *K* as PEGDA concentration is increased from 4.5 to 6.5%. On the contrary, as PEGDA content is increased to 10%, the consistency index increases substantially, suggesting that there is a complex interplay between alginate, CMC, and PEGDA that affects the shear-thinning behavior. All four formulations are shear-thinning fluids (*n* < 1) where the flow behavior index of each formulation remains relatively constant with increasing PEGDA concentration. Owing to the high strain experienced during extrusion-based 3DP, followed by a sharp reduction in shear rate to zero, the thixotropic behavior was evaluated via a three-interval thixotropic test (3ITT). The 3ITT imitates printing conditions and enables an assessment of work softening.

**Figure 2:**
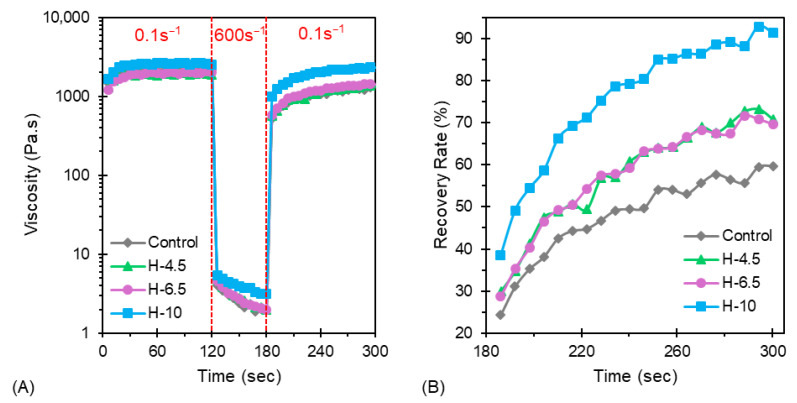
Thixotropic behavior of hybrid hydrogels as measured via (**A**) 3ITT and (**B**) corresponding recovery rates. All PEGDA-containing hydrogels exhibit high work softening and recovery of low-shear viscosity, thus indicating thixotropic behavior conducive for direct-write 3D bioprinting.

As shown in Figure 2A, the measured viscosity of all four formulations plateaus after approximately 50 s at 0.1 s^−1^. Additionally, the viscosity of H-10 is higher than that of the controls, H-4.5 and H-6.5. This is attributed to the increased PEGDA content, which has a high viscosity in comparison to H_2_O. Additionally, PEGDA may form hydrogen bonds with the alginate/CMC/H_2_O gel. The increased resistance to flow for H-10 may negatively impact cell viability during printing, as shear stresses will be greater than those of the other formulations examined. This would not be a concern for acellular scaffolds, where shape fidelity and mechanical properties are the main considerations. Notably, the recovery half-times varied with increasing PEGDA concentration (Figure 2B). Recovery half-time for the control sample is approximately 250 s for the control, 220 s for the H-4.5 and H-6.5 materials, and about 190 s for H-10. As shown, the control exhibits the lowest recovery rate of all materials tested, recovering 60% of its low shear viscosity. H-4.5 and H-6.5 recover approximately 70% of their low shear viscosity within 100 s. H-10 displayed the highest recovery rate, reaching 91% of its low shear viscosity within 100 s. The behavior of all PEGDA-containing hydrogels is conducive to shape retention following deposition, where H-4.5 and H-6.5 are expected to have slightly reduced shape retention and print resolution.

### 3.2. Printability of Unreacted Hybrid Hydrogels

**Figure 3:**
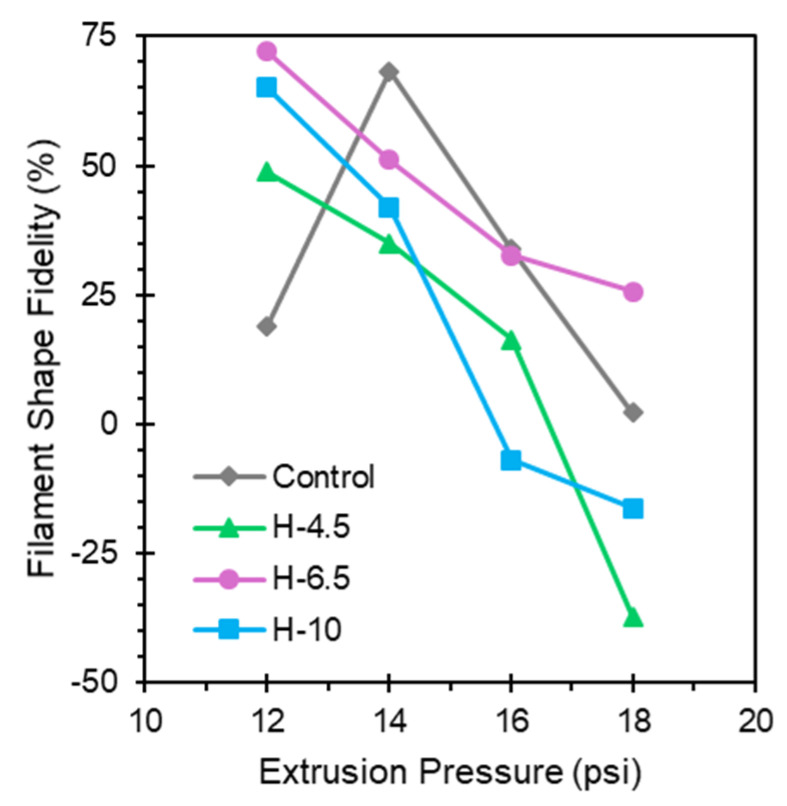
Filament shape fidelity test data indicates differences between filament widths as a function of extrusion pressure. H-6.5 exhibits the highest filament shape fidelity swelling across all tested extrusion pressures.

To further address the unreacted hybrid hydrogels’ filament uniformity, printability tests were carried out. Filament uniformity was tested via a four-line test for each formulation at extrusion pressures of 12–18 psi. These results are presented in Figure 3.

The balance between extrusion pressure and filament width affects print speed, print resolution, and cell viability during printing. Filament shape fidelity represents how closely hydrogel extrudates match the nozzle diameter. At high pressures, material flows more quickly, which necessitates increased print speeds; however, die swell results in increased filament widths, therefore reducing print resolution. Additionally, higher pressures place more internal stresses on the hydrogels, which negatively affect cell viability during printing. As shown in Figure 3, H-6.5 exhibits the best performance overall and the least sensitivity to changes in extrusion pressure. Pictures of filament shape fidelity tests for all formulations are shown in Supplemental Figure S3.

**Figure 4:**
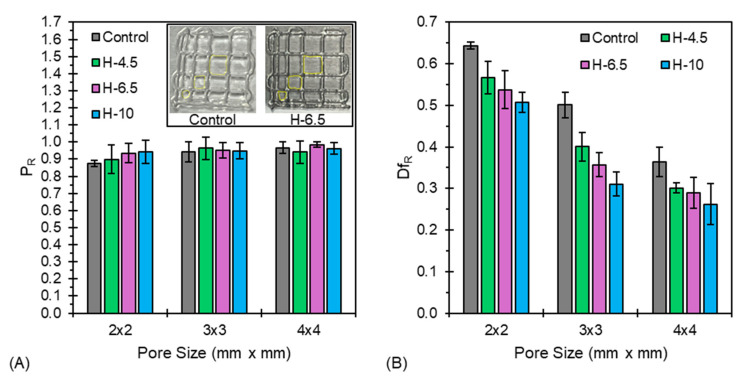
Filament fusion tests indicate the (**A**) printability, (inset) representative images of control and H-6.5 filament fusion tests with marked-up pore areas, and (**B**) diffusion rate of each formulation at various pore sizes.

Filament fusion tests rely on the generation of a bilayer scaffold with varying pore sizes (2 × 2, 3 × 3, and 4 × 4 mm) to evaluate printability (P_R_) and diffusion rate (Df_R_) for unreacted hybrid hydrogels via a single test. For the data presented in Figure 4, an extrusion pressure of 14 psi was utilized, as this provided a balance between filament continuity, print speed, and resolution. Supplemental Figure S4 depicts the methodology employed for this test.

As shown in Figure 4A, H-6.5 demonstrates the best printability across all tested pore sizes, boasting P_R_ = 1 for a 4 × 4 mm pore in the unreacted state. H-10 underperformed H-4.5 and H-6.5 for 3 × 3 and 4 × 4 pore sizes, matching the poor performance of the control sample. The high printability (P_R_ > 0.88) of all tested hydrogels across multiple pore sizes indicates that these materials are amenable to high-resolution direct-write (DW) 3D bioprinting, as the base formulation (A_4_C_4_) was down-selected from a previous study that evaluated alginate and CMC content [17]. It is important to mention that while P_R_ is a good indicator of shape fidelity of pore geometry and internal/external macro-porosity for large-scale (≥1 cm) 3D bioprinted scaffolds, this factor alone may not always be sufficient. In order to capture material-specific behaviors such as filament spread, Df_R_ was evaluated. Figure 4B indicates that the neat A_4_C_4_ (control) exhibits the highest diffusion rates across all tested pore sizes, and, therefore, the presence of PEGDA improves filament stability post-extrusion. Df_R_ decreases with increasing pore size. This is attributed to the decrease in filament width relative to larger pore areas. H-10 exhibited the lowest Df_R_ across all pore sizes, signifying that at 10% *w*/*v* PEGDA, the hydrogel filament spreads the slowest following extrusion. This result reflects the increase in viscosity as PEGDA content is increased, with H-10 exhibiting the highest viscosity of all tested formulations, as shown in Figure 2A.

To strengthen the quantitative rigor of our findings, a one-way ANOVA (see Supplemental Figures S5 and S6 and Table S1) was performed on the twelve printability conditions using the full dataset of P_R_ and Df_R_. The analysis demonstrated a significant effect of the printing condition on Df_R_ outcomes (F = 43.09, *p* < 0.0001), confirming that the observed trends are statistically meaningful rather than random variation. The model showed excellent explanatory power (R^2^ = 95.18%, adjusted R^2^ = 92.97%), indicating that the majority of the variability in DF is accounted for by the tested factors. Interval plots with 95% confidence levels further illustrate clear differences among groups, supporting our conclusion that the tested parameters, particularly nozzle size and extrusion pressure, produce measurable and statistically validated improvements in printability performance.

The results for P_R_ indicated no statistically significant differences among groups (F = 0.95, *p* = 0.511), and the model explained a modest portion of the variability (R^2^ = 30.40%). Although minor fluctuations were observed, the printability values for all conditions remained close to 1.0, demonstrating consistently high shape fidelity across formulations. The interval plot with 95% confidence intervals further supports that each bioink composition produced pores with near-ideal square geometry. These findings confirm that the observed printability trends are quantitatively stable.

While printability provides a useful measure of how closely a printed pore approximates a perfect square, this parameter alone does not fully capture the functional suitability of a bioink for extrusion-based bioprinting. A pore can exhibit a high printability value—even exactly 1.0—regardless of whether the pore is slightly smaller or larger than the intended dimension. In contrast, the diffusion rate more critically reflects the bioink’s ability to maintain dimensional accuracy, as it quantifies the degree of filament spreading immediately after extrusion. ANOVA on the diffusion dataset revealed statistically significant differences between formulations (F = 43.09, *p* < 0.0001), with a high explanatory power (R^2^ = 95.18%). These results highlight that the diffusion rate is far more sensitive to material composition and nozzle pressure than printability. A formulation with excellent printability but excessive diffusion will not maintain proper filament width, leading to structural inaccuracy, compromised pore geometry, and reduced reproducibility in multilayer constructs. Thus, although printability is an important indicator of overall geometric fidelity, diffusion rate serves as the more discriminating and functionally relevant criterion for assessing whether a bioink is truly suitable for high-resolution 3D bioprinting.

### 3.3. Reaction Kinetics

To guide parameter development for the DW 3D bioprinting of all hybrid hydrogels, photo-DSC and photorheological experiments were performed. Techniques used to measure reaction kinetics of free radical polymerization include Electron Spin Resonance (ESR) Spectroscopy [34,35], Fourier Transform Near-Infrared Spectroscopy [35], Gel Permeation Chromatography [35], Differential Scanning Calorimetry (DSC) [36,37], photo-DSC [38,39], photorheology [40,41,42], or real-time FTIR [40,41]. DSC is one of the most ubiquitous techniques for measuring reaction kinetics owing to its ease of use and ability to relate heat evolved during the reaction to the number of bonds formed [37,43,44,45]. Here, the degree of cure, or conversion (α), is determined by Equation (6).

(6) $$\alpha =\frac{\Delta H(t)}{{\Delta H}_{MAX}}\times 100$$

**Figure 5:**
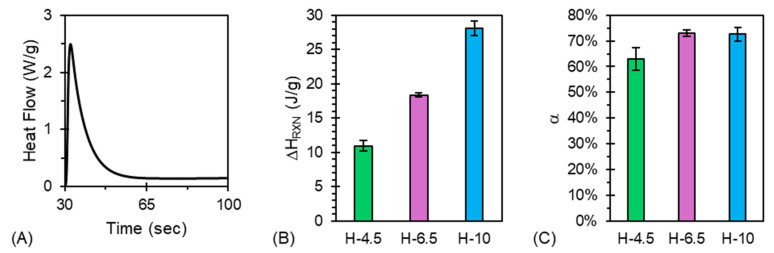
(**A**) Characteristic photo-DSC curve of H-6.5 during irradiation, (**B**) reaction enthalpy (∆H_RXN_) increases proportionally with PEGDA content, and (**C**) PEGDA conversion of all hybrid hydrogel formulations is above 60%.

Figure 5 provides reaction enthalpies and conversion data for the hybrid hydrogels tested.

A characteristic photo-DSC curve of H-6.5 is shown in Figure 5A. This curve indicates that the majority of the photo-crosslinking is complete after approximately 20 s of irradiation. This trend is present across all tested hydrogels, providing a good basis for the structural development of these materials in the presence of UV exposure post-extrusion. Characteristic photo-DSC curves for all tested materials are shown in Supplemental Figure S7. Due to the increase in reactive groups per mole of hydrogel solution, there is a sensible increase in the reaction enthalpy concomitant with PEGDA content, as shown in Figure 5B. Figure 5C indicates that conversion remains relatively insensitive to PEGDA concentration across the tested hydrogels. We attribute the low conversion of these materials to their characteristic gelled nature prior to irradiation. The high viscosity of the gels hinders the diffusion of PEGDA molecules during polymerization, reducing their ability to reach full conversion.

**Figure 6:**
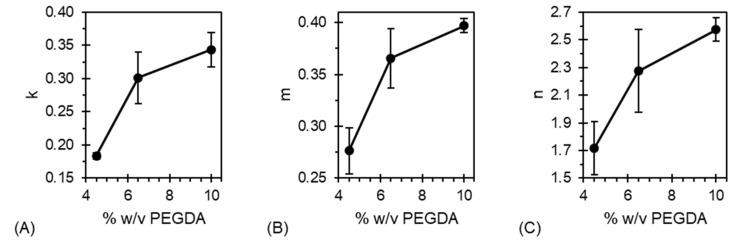
Kamal model parameters obtained by fitting Equation (7) to photo-DSC data. The (**A**) rate constant, (**B**) autocatalytic rate constant, and (**C**) reaction order all increase with PEGDA concentration for the hybrid hydrogels tested.

The phenomenological, autocatalytic model proposed by Kamal [45,46] is applied to describe the curing of a variety of thermosetting resins, including meth(acrylates), which exhibit auto-acceleration [38,44,45,47,48,49]. To understand differences in the reaction kinetics of the tested hydrogels as obtained via photo-DSC, the Kamal model was used (Equation (7)).

(7) $$\frac{d\alpha }{dt}=k\alpha^{m}{\left(1-\alpha \right)}^{n}$$

where (1 − α) corresponds to the concentration of reactants, *k* is the rate constant, and *m* and *n* are the reaction orders. Classical photopolymerizations rely on chain-growth initiated by free radicals; the major steps that occur during this process include initiation, propagation, and termination. Prior to initiation, the photoinitiator is decomposed to generate free radicals. Figure 6 portrays the bulk rate constant (*k*), autocatalytic rate constant (*m*), and reaction order (*n*) as a function of PEGDA content. Kamal model fits for all materials exhibited adjusted R^2^ values greater than 0.9 for H-4.5, and greater than 0.97 for H-6.5 and H-10. The high goodness of fit provides confidence in employing the Kamal model to capture the kinetic evolution of these systems.

The general increase in Kamal model parameters as PEGDA concentration increases is credited to the increase in reactive groups per mole of hydrogel solution. H-10 exhibits the highest values for *k*, *m*, and *n,* which indicates that the increased concentration of acrylate groups fuels faster reaction kinetics overall. Differences in the autocatalytic rate constant and reaction order (*m* and *n*, respectively) alter the auto-acceleration characteristics. As *m* decreases below *n*, auto-acceleration is hastened; conversely, if *n* decreases below *m*, auto-acceleration is hindered. It is therefore notable that *m* is lower than *n* for all prepared hydrogels. As more PEGDA is incorporated into the hydrogels, the gap between these parameters (i.e., *n* − *m*) demonstrates a marked increase, from 1.42 for H-4.5 to 2.16 for H-10. This indicates that auto-acceleration is hastened as PEGDA concentration increases. The hastening of auto-acceleration with PEGDA content could improve shape integrity during printing owing to crosslink density increases during irradiation.

**Figure 7:**
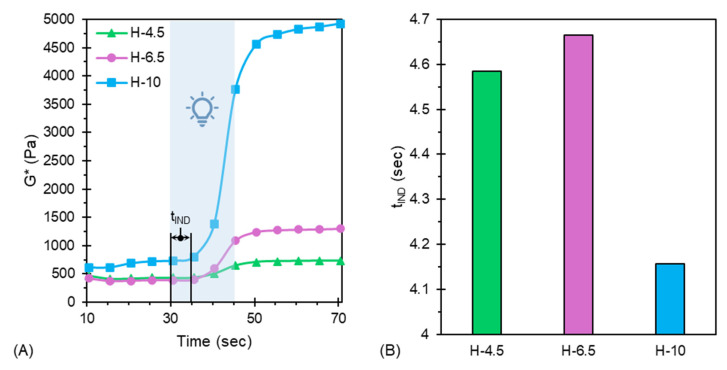
All samples irradiated at 365 nm (6 mW/cm^2^) for 15 s, as indicated by blue-gray highlighted section in (**A**) photorheological curves and the corresponding (**B**) induction time (t_IND_) of all tested hydrogels.

To further explain why these differences are manifest, photorheological experiments were performed. Photorheology captures polymerization kinetics occurring throughout transition regimes, which are rather complex in nature, with incredible pace over brief time scales. The conversion of acrylate groups during free radical polymerization generally follows a sigmoidal-shaped curve. The resulting behavior represents material moduli that change by five or six orders of magnitude in just a few seconds [50]. Due to the mobile restriction of termination reactions, auto-acceleration (Trommsdorff or gel effect) [51] is observed early on during polymerization, in alignment with a high kinetic chain length; this is indicated by the crossover of storage (G′) and loss (G″) moduli during photorheological experiments. As viscosity increases, a reduction in termination yields an increase in unreacted macroradicals, which are tethered to reacted polymer chains [51]. Tethering of most acrylate groups results in diffusion-controlled propagation, and auto-deceleration is typically observed. The resulting final double bond conversion (DBC) is therefore always some fraction of 100% due to the occlusion of both radicals and unreacted acrylate moieties [51,52]. Figure 7 provides an overview of characteristic photorheological curves and the induction time (t_IND_) for each of the hybrid hydrogels.

As shown in Figure 7A, the complex moduli (G*) of H-4.5 and H-6.5 are in close proximity prior to irradiation. The high G* of H-10 prior to irradiation matches the viscosity trends shown in Figure 2A. Following irradiation, a marked increase in G* is observed concurrent with PEGDA concentration. The range of material moduli obtained across the hybrid hydrogels (E = 620–4600 Pa) provides utility for the biomedical engineering of brain, kidney, and heart tissue [53]. Induction time reflects a lag in the onset of polymerization following the opening of the shutter during photorheological experiments. The high t_IND_ of these materials is attributed to the consumption of inhibitors contained within PEGDA in conjunction with the high viscosity of all hydrogels in the unreacted state. The high viscosity of these materials in their unreacted state hinders the ability of PEGDA monomers to diffuse and react. H-4.5 and H-6.5 have similar t_IND_; however, t_IND_ is reduced for H-10 owing to the higher concentration of PEGDA monomers per unit volume of this formulation. These results further suggest that sufficient irradiation during 3DP (5–15 s) would substantially increase the moduli of the tested hydrogels, thus enhancing their structural integrity.

**Figure 8:**
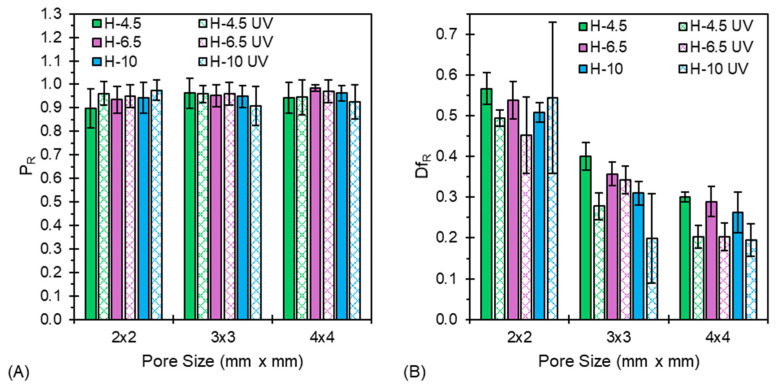
Filament fusion tests with and without UV irradiation indicate the (**A**) changes in printability, and (**B**) reduction in diffusion rate of each formulation at various pore sizes.

To understand the effect of UV irradiation on printability and diffusion rate of the hydrogels, filament fusion tests were run with the UV light on. Figure 8 compares P_R_ and Df_R_ of PEGDA-containing hydrogels with and without UV irradiation.

With respect to Figure 8A, P_R_ remains invariant or exhibits a small improvement with the presence of UV irradiation. As shown in Figure 8B, Df_R_ exhibits a general reduction across all formulations and pore sizes with the presence of UV irradiation. This decrease in diffusion rate is attributed to the increase in material moduli under UV irradiation and thus the arrest of filament diffusion post-extrusion. H-4.5 exhibits the most pronounced decrease in Df_R_ across all pore sizes with the presence of UV irradiation, owing to its low viscosity prior to irradiation. This agrees well with the increases in G* during photorheological experiments (Figure 7A). These results further indicate the importance of considering both diffusion rate and printability in evaluating the shape fidelity of printed constructs. Supplemental Table S2 provides metrics P_R_ and Df_R_, and Supplemental Figure S8 shows images of all filament fusion prints for the tested formulations.

**Figure 9:**
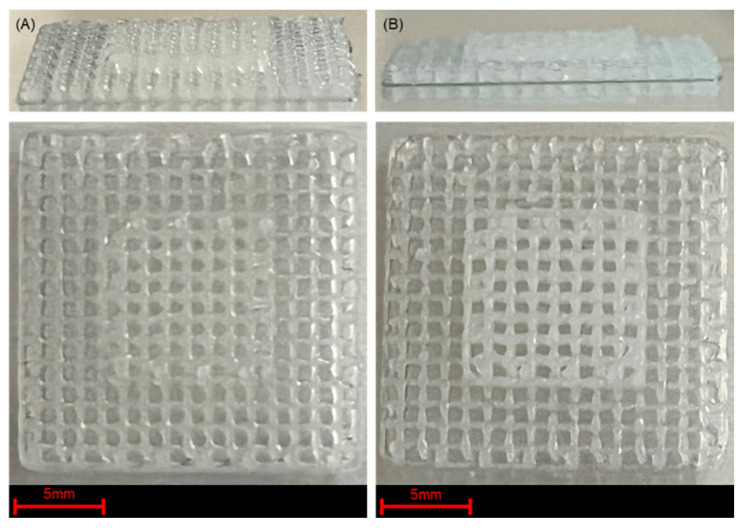
Shape fidelity improvement for 3D-printed scaffolds shown by isometric and top views of 10-layer free form constructs prepared utilizing (**A**) H-6.5 and (**B**) H-10.

Filament fusion results for the unreacted hydrogels indicated an inability to obtain small pore sizes (<4 mm^2^). As shown in the inset of Figure 4A, 1 × 1 mm pores were unable to be realized for the Control and H-6.5 samples owing to the diffusion of material post-extrusion. Therefore, in situ UV crosslinking was required to obtain 10-layer scaffold constructs, constructed with 1 × 1 mm pores as shown in Figure 9.

The representative scaffolds shown in Figure 9 highlight the ability to generate free-form structures with H-6.5 and H-10. Figure 9A supports the high performance of H-6.5, which exhibited the lowest diffusion rates and highest printability across all tested pore sizes, in addition to the lowest variations in filament shape fidelity over a wide range of extrusion pressures. These results provide promise in the role that UV irradiation plays in developing high-fidelity DW 3DP scaffolds. To further elucidate the biocompatibility of the tested hydrogels, cytotoxicity tests were performed.

### 3.4. Cytotoxicity Test

**Figure 10:**
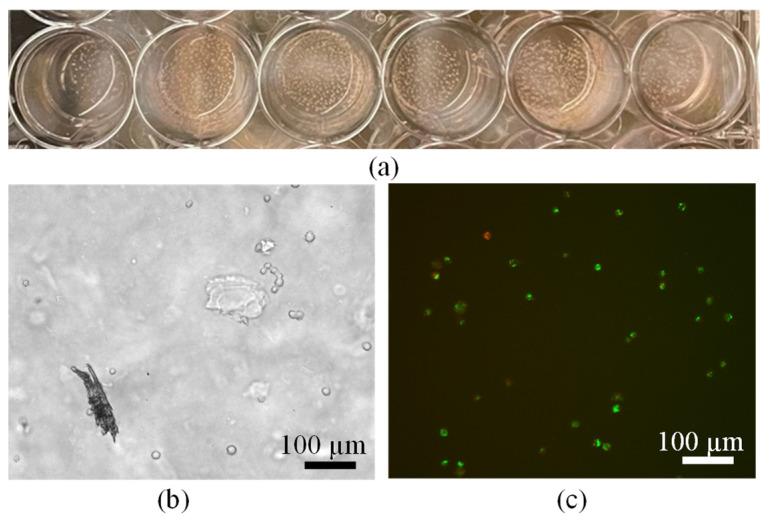
(**a**) An amount of 0.25 mL squirted out on top of cells, (**b**,**c**) live (green)/dead (red) assay after 24 h, observed more than 90% cell viability.

For the cells on standard tissue culture plastic (TCP) with hydrogel on top condition, human mesenchymal stem cells (MSCs) remained visible through the hydrogel layer and exhibited a rounded and compact morphology; however, this response is typical for cells interacting with softer or less adhesive biomaterial substrates [54]. This morphology is widely associated with culture on soft substrates or matrices with low integrin-binding ligand density, where reduced cell spreading and contractility lead to small, rounded cells or spheroids rather than the well-spread fibroblast-like phenotype seen on stiff, adhesive TCP [55]. Importantly, live/dead fluorescence imaging demonstrated more than 90% cell viability across all conditions after 24 h, confirming that the hydrogel—whether used as an overlay or as a direct seeding surface—is not cytotoxic and maintains strong short-term cytocompatibility. These results collectively indicate that although the hydrogel influences early cell spreading and adhesion dynamics, it provides a supportive environment capable of sustaining viable MSCs. While a 24 h time point provides only an initial indication of cytocompatibility, the evaluation will be extended to 3- and 7-day time points in future work to assess longer-term viability, proliferation, and cell–material interactions. To further enhance cell attachment, proliferation, and overall viability in future work, we recommend (i) optimizing hydrogel stiffness and crosslink density to more closely match MSC-preferred mechanical environments, which can significantly improve cell morphology and proliferation. (ii) Preconditioning or coating the hydrogel surface with serum proteins or extracellular matrix components before seeding to enhance initial attachment and reduce cell rounding. Representative micrographs for each experimental group are presented in Figure 10.

Previous studies, including our own, have shown that although pure alginate, CMC, and PEGDA individually do not provide strong proliferative support, blending alginate with CMC yields a synergistic improvement in cellular behavior. Specifically, our previous work demonstrated that alginate–CMC hydrogels support significantly higher cell viability than pure alginate at later culture time points [17,26]. Viability in the alginate–CMC group increased to approximately 80% on day 15 compared to ~72% in pure alginate, representing a statistically significant improvement (*p* < 0.05). By day 23, this difference became even more pronounced, with viability in alginate–CMC reaching ~86%, compared to ~80% in pure alginate. SEM analysis further revealed that incorporation of CMC increases structural porosity over time, which enhances nutrient and growth factor diffusion as well as waste removal [17]. This improved mass transport likely contributes to the superior viability observed in the 4% Alg–4% CMC cell-laden constructs. Moreover, our further study demonstrated that controlled modification of printing process parameters—particularly extrusion pressure—can raise the viability of alginate–CMC constructs to nearly 90%, highlighting the synergistic influence of both material formulation and bioprinting conditions on cell survival [26]. These findings are consistent with other recent reports demonstrating that CMC incorporation improves the bioactivity of alginate matrices and supports enhanced cellular proliferation [56,57]. While PEGDA itself is non-adhesive, several studies show that incorporating PEGDA into alginate-based composite or double-network hydrogels improves the overall mechanical stability and mass transport of the matrix, supporting high cell viability and sustained proliferation compared with single-component alginate systems, particularly in 3D bioprinting contexts [31,58].

## 4. Conclusions

This investigation provides a comprehensive assessment of the curing characteristics and direct-write 3D bioprintability of alginate and CMC-based hydrogels. By varying the concentration of a biocompatible photo-crosslinking agent, PEGDA, its effects on metrics such as filament swelling, diffusion rate, and printability were evaluated. PEGDA-containing hydrogels were compared with a control sample containing 4% *w*/*v* alginate and 4% *w*/*v* CMC. The incorporation of PEGDA resulted in a marked decrease in filament swelling percentage across 12–18 psi extrusion pressures in comparison to the control sample. This indicates an enhancement in the structural stability of filaments post-extrusion for hydrogels that are augmented with PEGDA. This was further supported by a decrease in diffusion rate (Df_R_) for unreacted H-4.5 and H-6.5 during filament fusion tests. The high printability (P_R_ > 0.88) of all unreacted hybrid hydrogels was attributed to the down-selection of the control formulation from a previous investigation [17]. Photo-DSC results were fitted to the Kamal model, indicating an increase in auto-acceleration (*n − m*) and rate constant (*k*) as the hydrogels were augmented with PEGDA. Photorheological results highlighted the range of material moduli obtained across the hybrid hydrogels (E = 620–4600 Pa), making them potentially appropriate for applications ranging from brain to heart tissue [53]. Although these results suggest that the materials could be useful in various biomedical applications, further studies—examining properties such as compressive modulus, swelling, and degradation in PBS, and effective diffusivity or permeability—are needed to validate their suitability for tissue engineering. Furthermore, the sufficiently fast curing kinetics of PEGDA-containing hydrogels enabled in situ UV crosslinking, which bolstered shape fidelity during DW 3D bioprinting. Substantial decreases in Df_R_ were observed when comparing filament fusion tests with and without UV irradiation. This was most pronounced for H-10, which was attributed to the crosslinking action of PEGDA, thus arresting the hydrogels’ post-extrusion. Ultimately, the high printability (P_R_ > 0.93) of all in situ UV irradiated hydrogels highlights their viability for high-resolution DW 3D bioprinting. This investigation of in situ UV crosslinking of hybrid hydrogels enables the development of biomaterials with a wide range of mechanical properties to enable the biomedical engineering of brain, kidney, and heart tissue.

## Figures and Tables

**Table 1:** Printing process parameters used in this article.

Construct	No. of Layers	Printing Process Parameters			
		Pressure (psi)	Speed (mm/s)	Intensity (mW/cm^2^)	Wavelength (nm)
Freeform	10	14	5	5	365

## Data Availability

The original contributions presented in the study are included in the article, further inquiries can be directed to the corresponding authors.

## Supplementary Materials

The following supporting information can be downloaded at: https://www.mdpi.com/article/10.3390/jfb17010030/s1, Figure S1: Chemical structures of materials used in this investigation; Figure S2: Custom in-house 3-axis “bedslinger” bioprinter, equipped with an in-situ dual (physical, chemical) crosslinking system; Figure S3: Filament shape fidelity test prints for (A) Control, (B) H-4.5, (C), H-6.5, and (D) H-10; Figure S4: (A) The fabricated scaffold for filament fusion follows a 0–90° pattern which captures the 2D effect and increasing filament to filament distance (FD). The range of FD used is 1–5 mm with 1 mm increments. After considering the filament diameter (d_f_), the raster width is defined as R_w_ = FD-d_f_. (B) Schematic illustration of two pores with identical outer dimensions but different actual pore areas (A_a_) due to material spreading, highlighting how shape fidelity can vary despite having the same printability value. (C) and (D) Optical images of two printed grids using different materials showing square-shaped pores with similar printability (P_R_ ≈ 1); however, (C) exhibits larger pore area and thinner filaments, while (D) shows reduced pore area and thicker filaments, indicating higher diffusion rates; Figure S5: Distribution of data for Df_R_; Figure S6: Distribution of data for P_R_; Figure S7: Characteristic photo-DSC curves for H-4.5, H-6.5, and H-10; Figure S8: Characteristic filament fusion test prints for (A) Control, (B) H-4.5, (C), H-6.5, and (D) H-10 with the UV light off, and (E) H-4.5, (F) H-6.5, (G) H-10 with the UV light on; Table S1: Comparison of factors for P_R_ and Df_R_; Table S2: Filament fusion test results for PEGDA containing hydrogels with and without UV irradiation.

## References

[1] Turnbull G., Clarke J., Picard F., Riches P., Jia L., Han F., Li B., Shu W. (2018). 3D bioactive composite scaffolds for bone tissue engineering. Bioact. Mater..

[2] Taniguchi D., Matsumoto K., Tsuchiya T., Machino R., Takeoka Y., Elgalad A., Gunge K., Takagi K., Taura Y., Hatachi G. (2018). Scaffold-free trachea regeneration by tissue engineering with bio-3D printing. Interact. Cardiovasc. Thorac. Surg..

[3] Agarwal S., Saha S., Balla V.K., Pal A., Barui A., Bodhak S. (2020). Current Developments in 3D Bioprinting for Tissue and Organ Regeneration–A Review. Front. Mech. Eng..

[4] Zhang Y.S., Haghiashtiani G., Hübscher T., Kelly D.J., Lee J.M., Lutolf M., McAlpine M.C., Yeong W.Y., Zenobi-Wong M., Malda J. (2021). 3D extrusion bioprinting. Nat. Rev. Methods Prim..

[5] Yu M., Yeow Y.J., Lawrence L., Claudio P.P., Day J.B., Salary R. (2021). Characterization of the Functional Properties of Polycaprolactone Bone Scaffolds Fabricated Using Pneumatic Micro-Extrusion. J. Micro Nano-Manuf..

[6] You F., Eames B.F., Chen X. (2017). Application of Extrusion-Based Hydrogel Bioprinting for Cartilage Tissue Engineering. Int. J. Mol. Sci..

[7] Nakayama K.H., Batchelder C.A., Lee C.I., Tarantal A.F. (2010). Decellularized Rhesus Monkey Kidney as a Three-Dimensional Scaffold for Renal Tissue Engineering. Tissue Eng. Part A.

[8] Mohammed A.A., Algahtani M.S., Ahmad M.Z., Ahmad J. (2021). Optimization of semisolid extrusion (pressure-assisted microsyringe)-based 3D printing process for advanced drug delivery application. Ann. 3D Print. Med..

[9] Ribeiro A., Blokzijl M.M., Levato R., Visser C.W., Castilho M., Hennink W.E., Vermonden T., Malda J. (2017). Assessing bioink shape fidelity to aid material development in 3D bioprinting. Biofabrication.

[10] Zhao F., Lacroix D., Ito K., van Rietbergen B., Hofmann S. (2020). Changes in scaffold porosity during bone tissue engineering in perfusion bioreactors considerably affect cellular mechanical stimulation for mineralization. Bone Rep..

[11] Kuo C.C., Qin H., Acuña D.F., Cheng Y., Jiang X., Shi X. (2019). Printability of Hydrogel Composites Using Extrusion-Based 3D Printing and Post-Processing with Calcium Chloride. J. Food Sci. Nutr..

[12] Diaz-Gomez L., Gonzalez-Prada I., Millan R., Da Silva-Candal A., Bugallo-Casal A., Campos F., Concheiro A., Alvarez-Lorenzo C. (2022). 3D printed carboxymethyl cellulose scaffolds for autologous growth factors delivery in wound healing. Carbohydr. Polym..

[13] Abdulmaged A.I., Soon C.F., Talip B.A., Zamhuri S.A.A., Mostafa S.A., Zhou W. (2022). Characterization of Alginate–Gelatin–Cholesteryl Ester Liquid Crystals Bioinks for Extrusion Bioprinting of Tissue Engineering Scaffolds. Polymers.

[14] Zhang Y., Liao J., Wang T., Sun W., Tong Z. (2018). Polyampholyte Hydrogels with pH Modulated Shape Memory and Spontaneous Actuation. Adv. Funct. Mater..

[15] Jia J., Richards D.J., Pollard S., Tan Y., Rodriguez J., Visconti R.P., Trusk T.C., Yost M.J., Yao H., Markwald R.R. (2014). Engineering alginate as bioink for bioprinting. Acta Biomater..

[16] Hurtado A., Aljabali A.A.A., Mishra V., Tambuwala M.M., Serrano-Aroca Á. (2022). Alginate: Enhancement Strategies for Advanced Applications. Int. J. Mol. Sci..

[17] Habib A., Sathish V., Mallik S., Khoda B. (2018). 3D Printability of Alginate-Carboxymethyl Cellulose Hydrogel. Materials.

[18] Rajabi M., McConnell M., Cabral J., Ali M.A. (2021). Chitosan hydrogels in 3D printing for biomedical applications. Carbohydr. Polym..

[19] Liu Q., Li Q., Xu S., Zheng Q., Cao X. (2018). Preparation and Properties of 3D Printed Alginate–Chitosan Polyion Complex Hydrogels for Tissue Engineering. Polymers.

[20] Flaibani M., Luni C., Sbalchiero E., Elvassore N. (2009). Flow cytometric cell cycle analysis of muscle precursor cells cultured within 3D scaffolds in a perfusion bioreactor. Biotechnol. Prog..

[21] Ramasamy S., Davoodi P., Vijayavenkataraman S., Teoh J.H., Thamizhchelvan A.M., Robinson K.S., Wu B., Fuh J.Y., DiColandrea T., Zhao H. (2021). Optimized construction of a full thickness human skin equivalent using 3D bioprinting and a PCL/collagen dermal scaffold. Bioprinting.

[22] Yoon Y., Kim C.H., Lee J.E., Yoon J., Lee N.K., Kim T.H., Park S.-H. (2019). 3D bioprinted complex constructs reinforced by hybrid multilayers of electrospun nanofiber sheets. Biofabrication.

[23] Luo W., Song Z., Wang Z., Wang Z., Li Z., Wang C., Liu H., Liu Q., Wang J. (2020). Printability Optimization of Gelatin-Alginate Bioinks by Cellulose Nanofiber Modification for Potential Meniscus Bioprinting. J. Nanomater..

[24] Lewis P.L., Green R.M., Shah R.N. (2018). 3D-printed gelatin scaffolds of differing pore geometry modulate hepatocyte function and gene expression. Acta Biomater..

[25] Jiang Y., Zhou J., Feng C., Shi H., Zhao G., Bian Y. (2020). Rheological behavior, 3D printability and the formation of scaffolds with cellulose nanocrystals/gelatin hydrogels. J. Mater. Sci..

[26] Habib M.A., Khoda B. (2022). Rheological Analysis of Bio-ink for 3D Bio-printing Processes. J. Manuf. Process..

[27] Lima T.d.P.L., Canelas C.A.D.A., Concha V.O.C., da Costa F.A.M., Passos M.F. (2022). 3D Bioprinting Technology and Hydrogels Used in the Process. J. Funct. Biomater..

[28] Shapiro J.M., Oyen M.L. (2014). Viscoelastic analysis of single-component and composite PEG and alginate hydrogels. Acta Mech. Sin..

[29] Xu H., Casillas J., Krishnamoorthy S., Xu C. (2020). Effects of Irgacure 2959 and lithium phenyl-2,4,6-trimethylbenzoylphosphinate on cell viability, physical properties, and microstructure in 3D bioprinting of vascular-like constructs. Biomed. Mater..

[30] de la Fuente A.Z., García-García A., Pérez-Álvarez L., Moreno-Benítez I., Larrea-Sebal A., Martin C., Vilas-Vilela J.L. (2024). Evaluation of Various Types of Alginate Inks for Light-Mediated Extrusion 3D Printing. Polymers.

[31] Greco I., Machrafi H., Iorio C.S. (2024). Double-Network Hydrogel 3D BioPrinting Biocompatible with Fibroblast Cells for Tissue Engineering Applications. Gels.

[32] Sevimli G., Kus E., Baran G., Marashian M., Tabatabaei N., Mustafaoglu N. (2025). Graphene nanoplatelets enhance neuronal differentiation of human bone marrow mesenchymal stem cells. Biol. Res..

[33] Jessop Z.M., Al-Sabah A., Gao N., Kyle S., Thomas B., Badiei N., Hawkins K., Whitaker I.S. (2019). Printability of pulp derived crystal, fibril and blend nanocellulose-alginate bioinks for extrusion 3D bioprinting. Biofabrication.

[34] Yamada B., Kageoka M., Otsu T. (1991). Dependence of propagation and termination rate constants on conversion for the radical polymerization of styrene in bulk as studied by ESR spectroscopy. Macromolecules.

[35] Zetterlund P.B., Yamazoe H., Yamada B., Hill D.J.T., Pomery P.J. (2001). High-Conversion Free-Radical Bulk Polymerization of Styrene: Termination Kinetics Studied by Electron Spin Resonance, Fourier Transform Near-Infrared Spectroscopy, and Gel Permeation Chromatography. Macromolecules.

[36] Achilias D.S. (2014). Investigation of the radical polymerization kinetics using DSC and mechanistic or isoconversional methods. J. Therm. Anal. Calorim..

[37] Harkous A., Colomines G., Allanic N., Mousseau P., Deterre R. (2013). Thermo-Kinetic Analysis of Liquid Silicone Rubber. Key Eng. Mater..

[38] Bakhshi H., Kuang G., Wieland F., Meyer W. (2022). Photo-Curing Kinetics of 3D-Printing Photo-Inks Based on Urethane-Acrylates. Polymers.

[39] DeRosa M.E., Baker L.S., Melock T.L., Yang B. (2021). Ultraviolet cure kinetics of a low Tg polyurethane acrylate network under varying light intensity and exposure time. Prog. Org. Coatings.

[40] Gorsche C., Harikrishna R., Baudis S., Knaack P., Husar B., Laeuger J., Hoffmann H., Liska R. (2017). Real Time-NIR/MIR-Photorheology: A Versatile Tool for the in Situ Characterization of Photopolymerization Reactions. Anal. Chem..

[41] Piguet-Ruinet F., Love B.J. (2007). Dynamic photorheological analysis of photopolymerizable urethane dimethacrylate resins with varying diluent content and light fluence. J. Appl. Polym. Sci..

[42] Achilias D.S., Tsagkalias I.S. (2018). Investigation of radical polymerization kinetics of poly(ethylene glycol) methacrylate hydrogels via DSC and mechanistic or isoconversional models. J. Therm. Anal. Calorim..

[43] Zhao L., Hu X. (2010). Autocatalytic curing kinetics of thermosetting polymers: A new model based on temperature dependent reaction orders. Polymer.

[44] Domínguez J., Alonso M., Oliet M., Rojo E., Rodríguez F. (2010). Kinetic study of a phenolic-novolac resin curing process by rheological and DSC analysis. Thermochim. Acta.

[45] Kamal M.R., Sourour S. (1973). Kinetics and thermal characterization of thermoset cure. Polym. Eng. Sci..

[46] Kamal M.R. (1974). Thermoset characterization for moldability analysis. Polym. Eng. Sci..

[47] Lee E.J., Park H.J., Kim S.M., Lee K.Y. (2018). Effect of Azo and Peroxide Initiators on a Kinetic Study of Methyl Methacrylate Free Radical Polymerization by DSC. Macromol. Res..

[48] Harkous A., Colomines G., Leroy E., Mousseau P., Deterre R. (2016). The kinetic behavior of Liquid Silicone Rubber: A comparison between thermal and rheological approaches based on gel point determination. React. Funct. Polym..

[49] Lang M., Hirner S., Wiesbrock F., Fuchs P. (2022). A Review on Modeling Cure Kinetics and Mechanisms of Photopolymerization. Polymers.

[50] Achilias D.S. (2007). A Review of Modeling of Diffusion Controlled Polymerization Reactions. Macromol. Theory Simul..

[51] Ligon-Auer S.C., Schwentenwein M., Gorsche C., Stampfl J., Liska R. (2015). Toughening of photo-curable polymer networks: A review. Polym. Chem..

[52] Wenand M., McCormick A.V. (2000). A Kinetic Model for Radical Trapping in Photopolymerization of Multifunctional Monomers. Macromolecules.

[53] Handorf A.M., Zhou Y., Halanski M.A., Li W.-J. (2015). Tissue Stiffness Dictates Development, Homeostasis, and Disease Progression. Organogenesis.

[54] Chaudhuri O., Gu L., Klumpers D., Darnell M., Bencherif S.A., Weaver J.C., Huebsch N., Lee H.-P., Lippens E., Duda G.N. (2015). Hydrogels with tunable stress relaxation regulate stem cell fate and activity. Nat. Mater..

[55] Trappmann B., Gautrot J.E., Connelly J.T., Strange D.G., Li Y., Oyen M.L., Cohen Stuart M.A., Boehm H., Li B., Vogel V. (2012). Extracellular-matrix tethering regulates stem-cell fate. Nat. Mater..

[56] Ozden A.K. (2022). Designing of Alginate-Based Tissue Scaffolds and Their Use in Mesenchymal Stem Cell Culture. Lokman Hekim Health Sci..

[57] Mahheidari N., Zamani S., Khademi R., Farahani M.K., Molzemi S., Salehi M. (2025). Biological macromolecules Alginate-Carboxymethyl Cellulose (Alg-CMC) hydrogel loaded with Botulinum toxin type A for skin wound treatment and functional tissue regeneration. Int. J. Biol. Macromol..

[58] Raus R.A., Nawawi W.M.F.W., Nasaruddin R.R. (2021). Alginate and alginate composites for biomedical applications. Asian J. Pharm. Sci..

