# Supplementary material for: Photo Crosslinkable Hybrid Hydrogels for High Fidelity Direct Write 3D Printing: Rheology, Curing Kinetics, and Bio-Scaffold Fabrication
Source: J Funct Biomater. 2026 Jan 4;17(1):30. doi: 10.3390/jfb17010030 (PMC12842536; doi:10.3390/jfb17010030)
Supplement: Supplementary file 1 [file jfb-17-00030-s001.zip › jfb-3995733-supplementary.pdf]

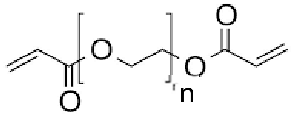

PEG diacrylate (PEGDA) (Mn=400 g/mol)

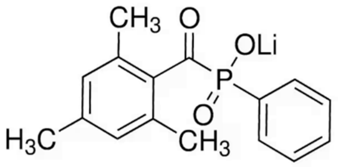

Lithium phenyl-2,4,6-trimethylbenzoylphosphinate (LAP)

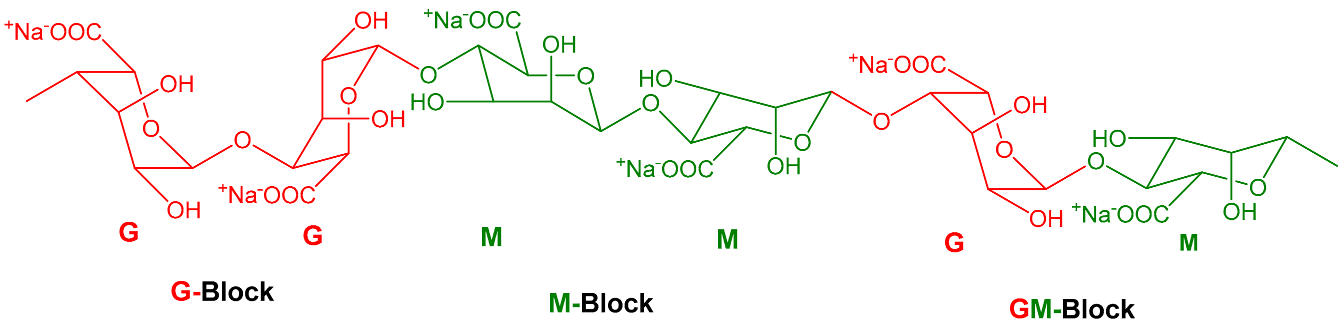

Alginate

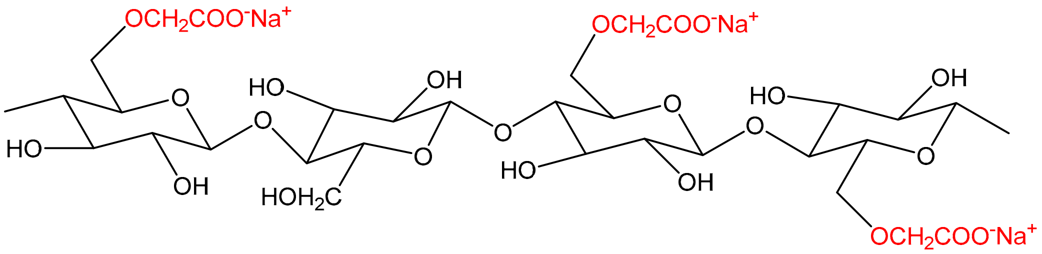

Carboxymethyl cellulose (CMC)

Figure S1: Chemical structures of materials used in this investigation.

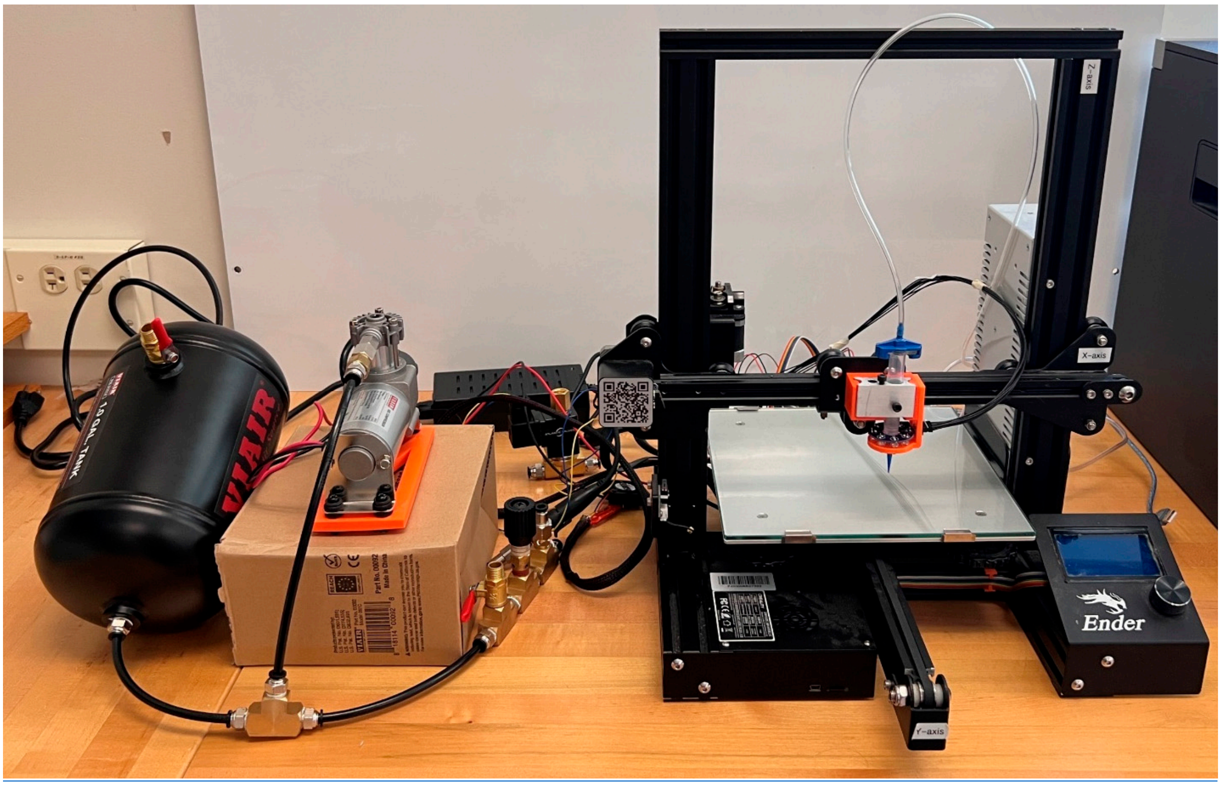

Figure S2: Custom in-house 3-axis "bedslinger" bioprinter, equipped with an in-situ dual (physical, chemical) crosslinking system.

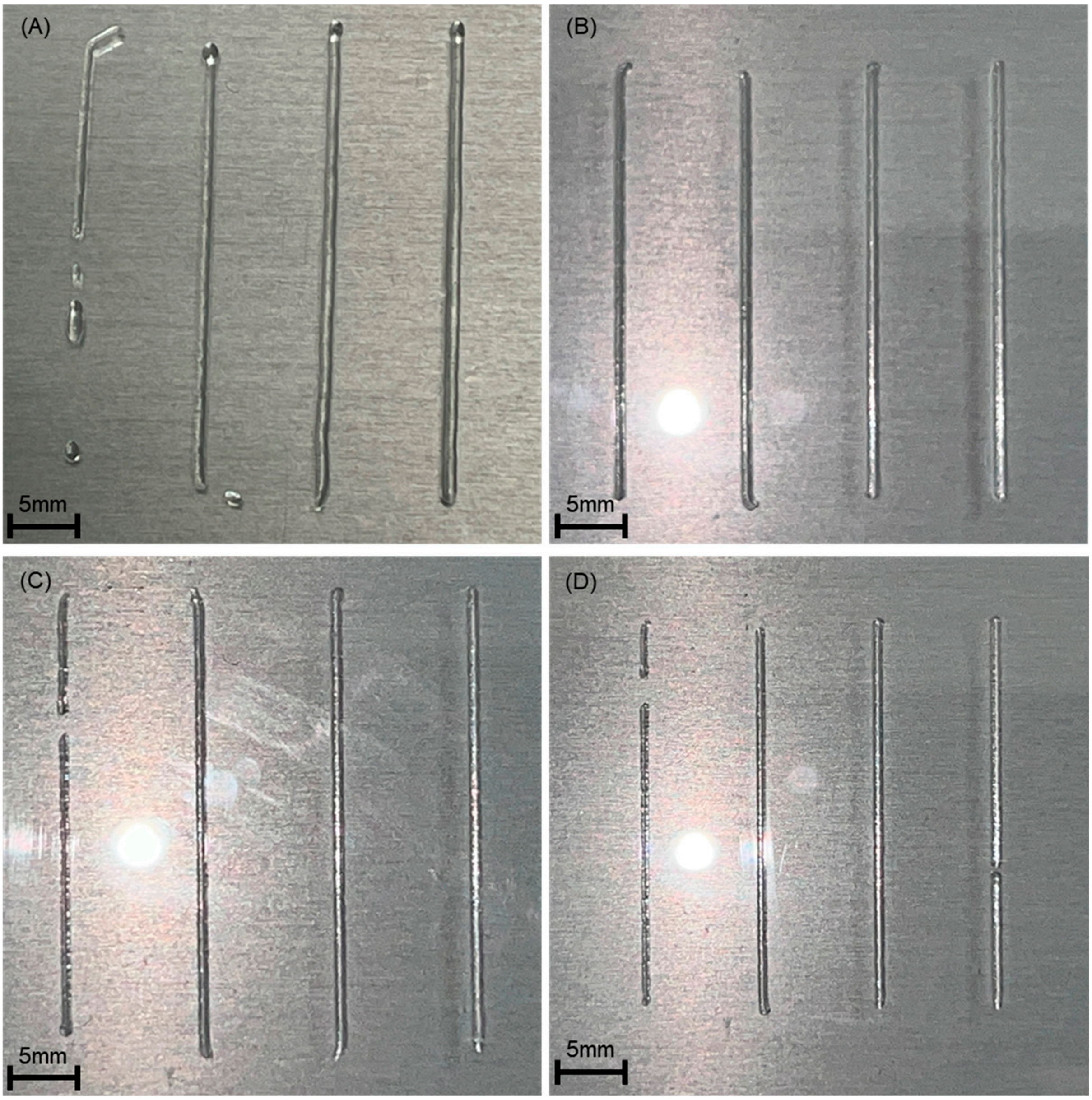

Figure S3: Filament shape fidelity test prints for (A) Control, (B) H-4.5, (C), H-6.5, and (D) H-10.

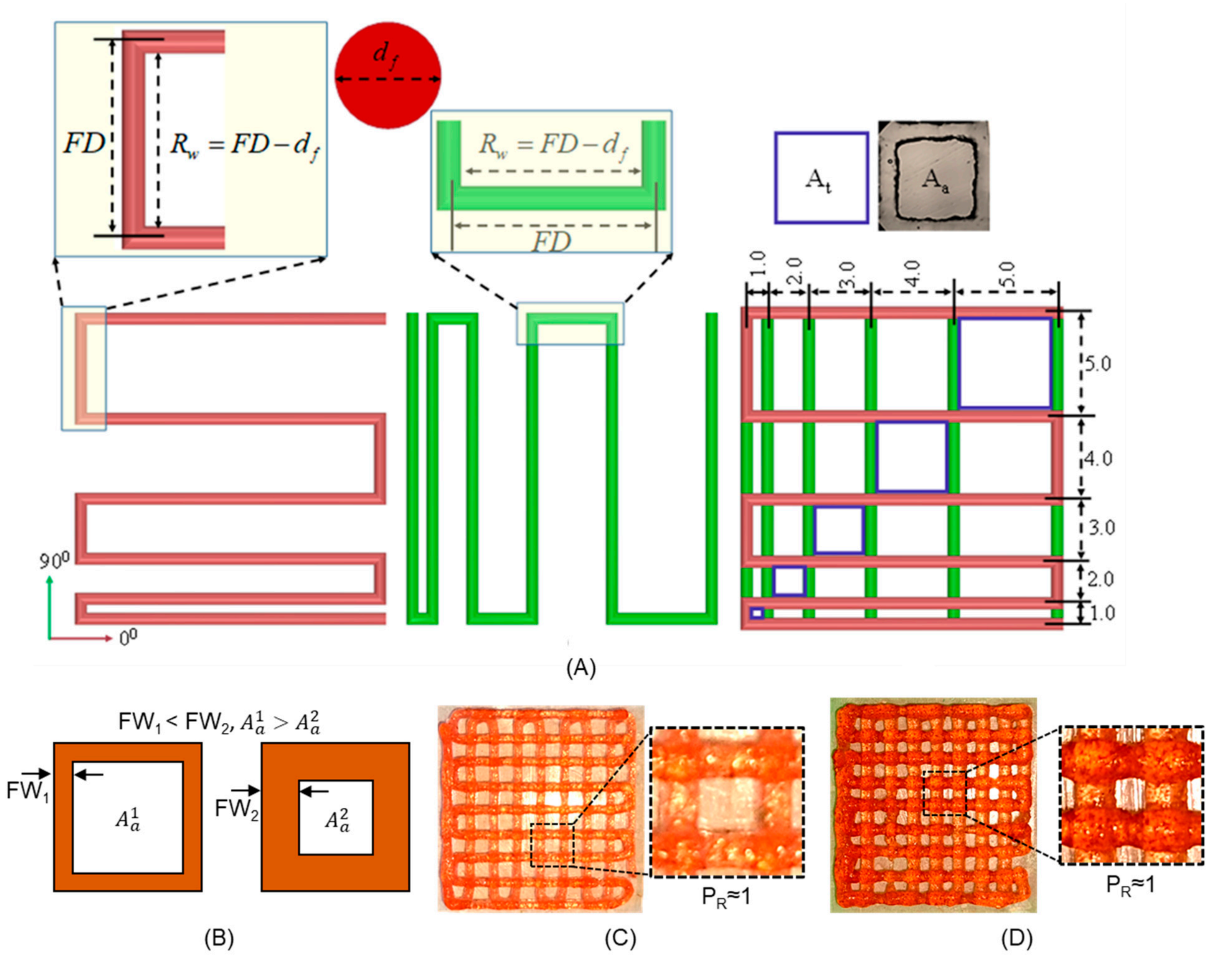

Figure S4: (A) The fabricated scaffold for filament fusion follows a 0° – 90° pattern which captures the 2D effect and increasing filament to filament distance (FD). The range of FD used is 1 – 5mm with 1mm increments. After considering the filament diameter (d_f), the raster width is defined as R_w=FD-d_f. (B) Schematic illustration of two pores with identical outer dimensions but different actual pore areas (A_a) due to material spreading, highlighting how shape fidelity can vary despite having the same printability value. (C) and (D) Optical images of two printed grids using different materials showing square-shaped pores with similar printability (P_R≈1); however, (C) exhibits larger pore area and thinner filaments, while (D) shows reduced pore area and thicker filaments, indicating higher diffusion rates.

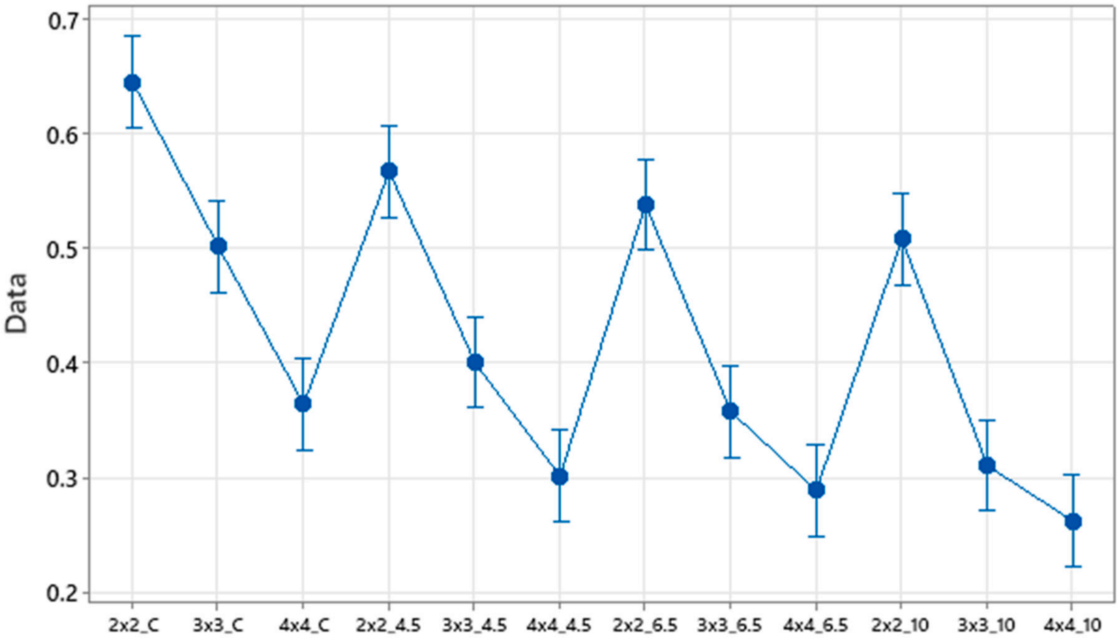

Figure S5: Distribution of data for Df_R.

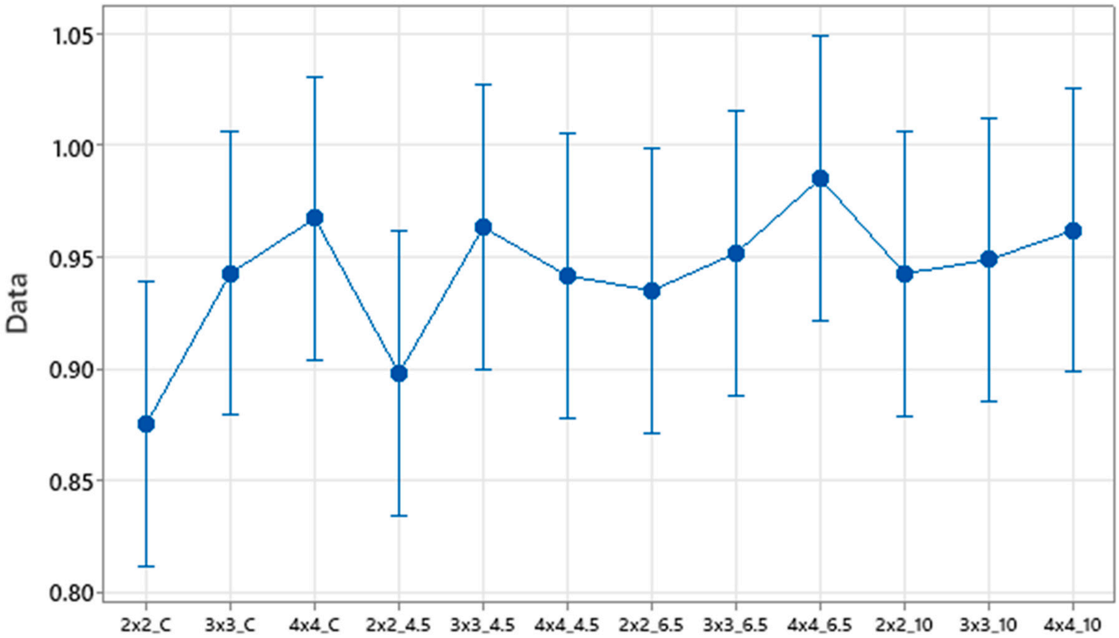

Figure S6: Distribution of data for P_R.

Table S1: Comparison of factors for P_R and Df_R.

Parameter	Diffusion Rate (Filament Spreading)	Printability (Pore Shape Accuracy)
DF (Factor)	11	11
Adjusted SS (Factor)	0.52523	0.02971
Adjusted MS (Factor)	0.047748	0.002701
F-value	43.09	0.95
P-value	0.000 (significant)	0.511 (not significant)
Model S	0.033288	0.053235
R-sq	95.18%	30.40%
R-sq (adj)	92.97%	0.00%
R-sq (pred)	89.16%	0.00%
Interpretation	Strong statistical differences between groups; diffusion rate strongly affect ed by bioink composition and printin g parameters.	No statistically meaningful differences betwe en groups; printability values remain consiste ntly high across all conditions.

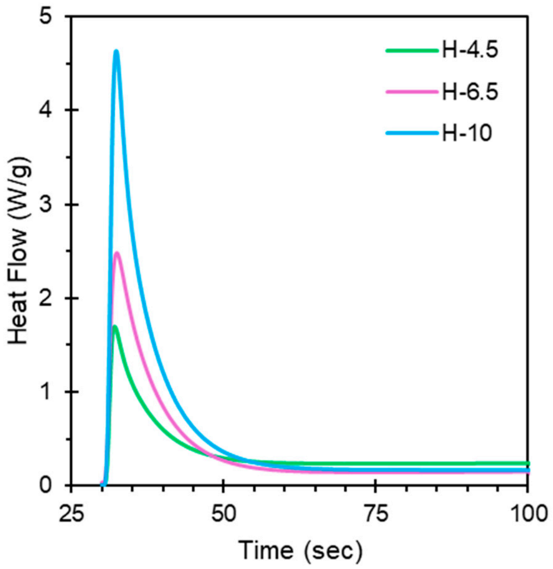

Figure S7: Characteristic photo-DSC curves for H-4.5, H-6.5, and H-10.

Table S2: Filament fusion test results for PEGDA containing hydrogels with and without UV irradiation.

Materials	Metrics	Df_R 2x2	Df_R 3x3	Df_R 4x4	P_R 2x2	P_R 3x3	P_R 4x4
H-4.5	UV off	0.57	0.40	0.30	0.90	0.96	0.94
	UV on	0.49	0.28	0.20	0.96	0.96	0.94
H-6.5	UV off	0.54	0.36	0.29	0.93	0.95	0.98
	UV on	0.45	0.34	0.20	0.95	0.96	0.97
H-10	UV off	0.51	0.31	0.26	0.94	0.95	0.96
	UV on	0.54	0.20	0.19	0.98	0.91	0.93

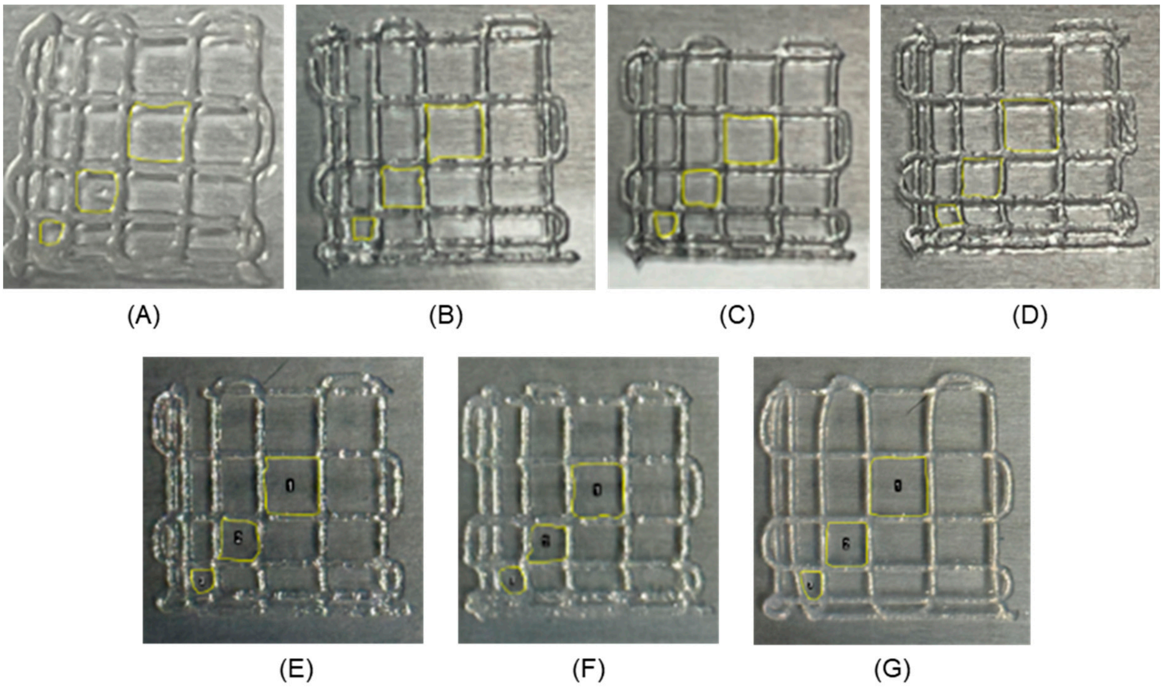

Figure S8: Characteristic filament fusion test prints for (A) Control, (B) H-4.5, (C), H-6.5, and (D) H-10 with the UV light off, and (E) H-4.5, (F) H-6.5, (G) H-10 with the UV light on.
